# The Influence of Baseline Hemoglobin A1c on Digital Health Coaching Outcomes in Adults With Type 2 Diabetes: Real-World Retrospective Cohort Study

**DOI:** 10.2196/24981

**Published:** 2021-06-16

**Authors:** Megan Martin, Jonathan Patterson, Matt Allison, Blakely B O’Connor, Dhiren Patel

**Affiliations:** 1 Medical Affairs Pack Health, LLC Birmingham, AL United States; 2 University of Alabama at Birmingham Birmingham, AL United States

**Keywords:** type 2 diabetes, mobile health, digital health coaching, digital therapy, diabetes support program, hemoglobin A1c, body mass index, diabetes distress

## Abstract

**Background:**

Digital health coaching is an increasingly common diabetes self-management support strategy for individuals with type 2 diabetes and has been linked to positive mental and physical health outcomes. However, the relationship between baseline risk and outcomes is yet to be evaluated in a real-world setting.

**Objective:**

The purpose of this real-world study was to evaluate trends in digital health coaching outcomes by baseline hemoglobin A_1c_ (HbA_1c_) to better understand which populations may experience the greatest clinical and psychosocial benefit.

**Methods:**

A retrospective cohort study design was used to evaluate program effect in a convenience sample of participants in a 12-week digital health coaching program administered by Pack Health. Participants were referred through their health care provider, payer, or employer. The program included patient-centered lifestyle counseling and psychosocial support delivered via telephone, text, and/or email. Self-reported HbA_1c_ and weight were collected at baseline and completion. Physical and mental health were assessed using the Patient-Reported Outcomes Measurement Information System (PROMIS) Global Health Short Form and the Diabetes Distress Scale-2. Changes in HbA_1c_, weight, BMI, and physical and mental health were analyzed within three participant cohorts stratified by baseline HbA_1c_ level.

**Results:**

Participants with complete HbA_1c_ data sets (n=226) were included in the analysis. The sample population was 71.7% (162/226) female, with 61.5% (139/226) identifying as white and 34.1% (77/226) as black. Most participants (184/226, 81.4%) reported a baseline HbA_1c_ ≥7%, and 20.3% (46/226) were classified as high risk (HbA_1c_ >9%). Across HbA_1c_ cohorts, the mean baseline BMI was 35.83 (SD 7.79), and the moderate-risk cohort (7% ≤ HbA_1c_ ≤ 9%) reported the highest mean value (36.6, SD 7.79). At 12 weeks, patients reported a significant decrease in HbAlc, and high-risk participants reduced their levels by the greatest margin (2.28 points; *P*<.001). Across cohorts, BMI improved by 0.82 (*P*<.001), with the moderate-risk cohort showing the greatest reduction (−0.88; *P*<.001). Overall, participants reported significant improvements for PROMIS scores, with the greatest change occurring in the high-risk cohort for whom physical health improved 3.84 points (*P*<.001) and mental health improved 3.3 points (*P*<.001). However, the lowest-risk cohort showed the greatest improvements in diabetes distress (−0.76; *P*=.005).

**Conclusions:**

Acknowledging the limitations in this real-world study design, the results reported here suggest that adults with type 2 diabetes with a high baseline HbA_1c_ or high BMI may benefit the most from patient-centered digital health coaching programs when compared to their lower risk counterparts. While all participants improved in physical and mental health categories, participants with high HbA_1c_ experienced the greatest HbA_1c_ reduction and individuals with the highest baseline BMI lost the most weight. These results may be used to inform referrals for patients who are more likely to benefit from digital health coaching.

## Introduction

### Background

Diabetes is a significant health concern in the United States, with an estimated 34.2 million Americans or 10.5% of the US population diagnosed with this condition [1]. More than 90% of those cases represent individuals with type 2 diabetes mellitus (T2DM), who face an increased risk of vascular health complications [2] in addition to substantial mental health burden [3-6]. Active diabetes self-management, healthy lifestyle behaviors, and improved psychosocial wellness have been associated with positive outcomes and a reduced risk of complications [7-10]. However, it is difficult for patients to learn about and sustain recommended changes given commonly faced barriers, including complex diabetes treatment plans [11], limited time during provider visits [12], and disparate access to education and community support resources [13-15]. Patient-centered strategies that address these barriers and provide sustained support are needed to drive positive behavioral, psychosocial, and clinical impacts [16-18].

Digital diabetes health coaching aligns with this need by complementing clinical care and education through individualized and ongoing support, which is delivered in an easily accessible format such as telephone, text, and email [19-24]. Randomized controlled trials have shown that digital diabetes health coaching programs have a positive impact on hemoglobin A_1c_ (HbA_1c_), weight, BMI, and mental health [25-29]. However, the degree and significance of the impact is variable. For example, a recent systematic review of digital diabetes interventions reported HbA_1c_ reductions, resulting from mobile coaching, ranging from 0.40% to 1.9% [30]. The highest impact intervention targeted patients with a baseline HbA_1c_ >9.0% and provided participants with a mobile app, web portal, and physician report [26], while the lowest impact intervention targeted individuals with an average HbA_1c_ of 6.86% and offered a mobile app, a web portal, and electronic health record integration [31]. Similarly, a 2013 study of a phone-based peer coaching program (N=299) showed variance in outcomes by baseline psychosocial and behavioral patient characteristics [32]. Specifically, the study reported a larger effect on lowering HbA_1c_ in patients with low levels of medication adherence and self-management support when compared to patients with higher levels of adherence and support [32].

While these studies have demonstrated the impact of digital diabetes health coaching under controlled research settings, it is not clear how these findings translate to real-world practice, which can be influenced by patient experience and utilization [33,34]. Real-world observations become particularly important in the consideration of digital health coaching, which often aims to drive psychosocial and behavioral change beyond the clinical and research settings. Moreover, digital health coaching programs are rapidly scaling across the United States, making the understanding of their real-world application increasingly important.

To optimize the scaling of digital health coaching for patients with diabetes and allocate care in the most equitable and efficient way, it is important to understand which patients may benefit the most. While HbA_1c_ is widely accepted as the benchmark for assessing glycemic control and risk [35-37], it is currently unclear how baseline HbA_1c_ modifies trends in digital health coaching outcomes. By understanding the impact of baseline HbA_1c_ on real-world digital health coaching outcomes, providers can make more informed referrals for patient participation in such programs.

### Objective

To build on the existing evidence base, this retrospective analysis examined real-world patient-reported data to evaluate the impact of a 12-week patient-centered digital diabetes health coaching program on glycemic control, BMI, weight, diabetes distress, and overall physical and mental health. Trends in outcomes were stratified by baseline glycemic risk, as assessed by HbA_1c_. We hypothesized that individuals with the highest baseline glycemic risk would experience the greatest improvements in mental and physical health outcomes.

## Methods

### Intervention Overview

The diabetes intervention under investigation was a multichannel diabetes support program, developed and delivered by Pack Health. The program, which is currently listed on the American Diabetes Association (ADA) peer-reviewed *Diabetes Support Directory* [38]*,* is designed to meet the ADA support programming criteria and align with the *Standards of Medical Care in Diabetes* [18]. It aligns with the *Standards* in multiple ways including, but not limited to, the provision of individualized psychosocial support and evidence-based behavioral modification strategies (eg, goal setting, motivational interviewing, problem solving) [18,39-42]. In accordance with ADA guidance for support programming [38], it aims to complement clinical care and education in an easily accessible format. To facilitate participant accessibility, Pack Health combines one-to-one phone-based health coaching with digital education and prompts via SMS text messaging and/or email, which can be accessed anywhere and at any time.

Coaches are allied health care professionals who complete a range of certification programs including, but not limited to, the American Association of Diabetes Care & Education Specialists (ADCES) Career Path Certificate Program [43], Centers for Disease Control and Prevention (CDC) Lifestyle Coach Training [44], and National Board for Health & Wellness Coaching (NBHWC) program [45]. Coaches also receive ongoing professional training on care escalation, health literacy, financial health, and cultural sensitivity. A multidisciplinary advisory team of physicians, pharmacists, and nurses provide programmatic oversight and quality assurance.

#### Intervention Process

**Figure 1:**
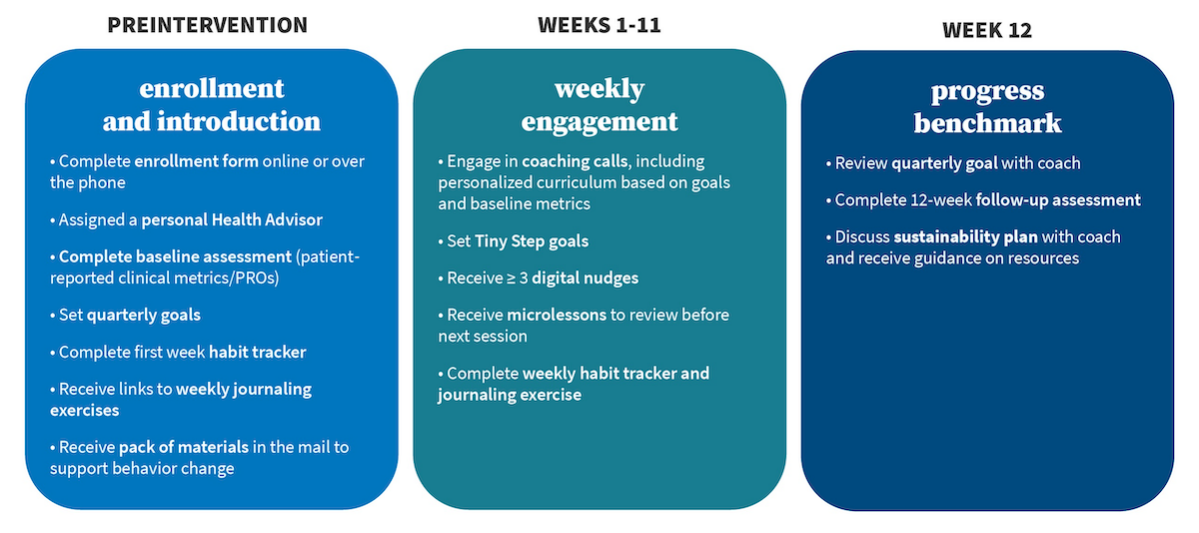
Program process: preprogram through week 12. PRO: patient-reported outcome.

**Figure 2:**
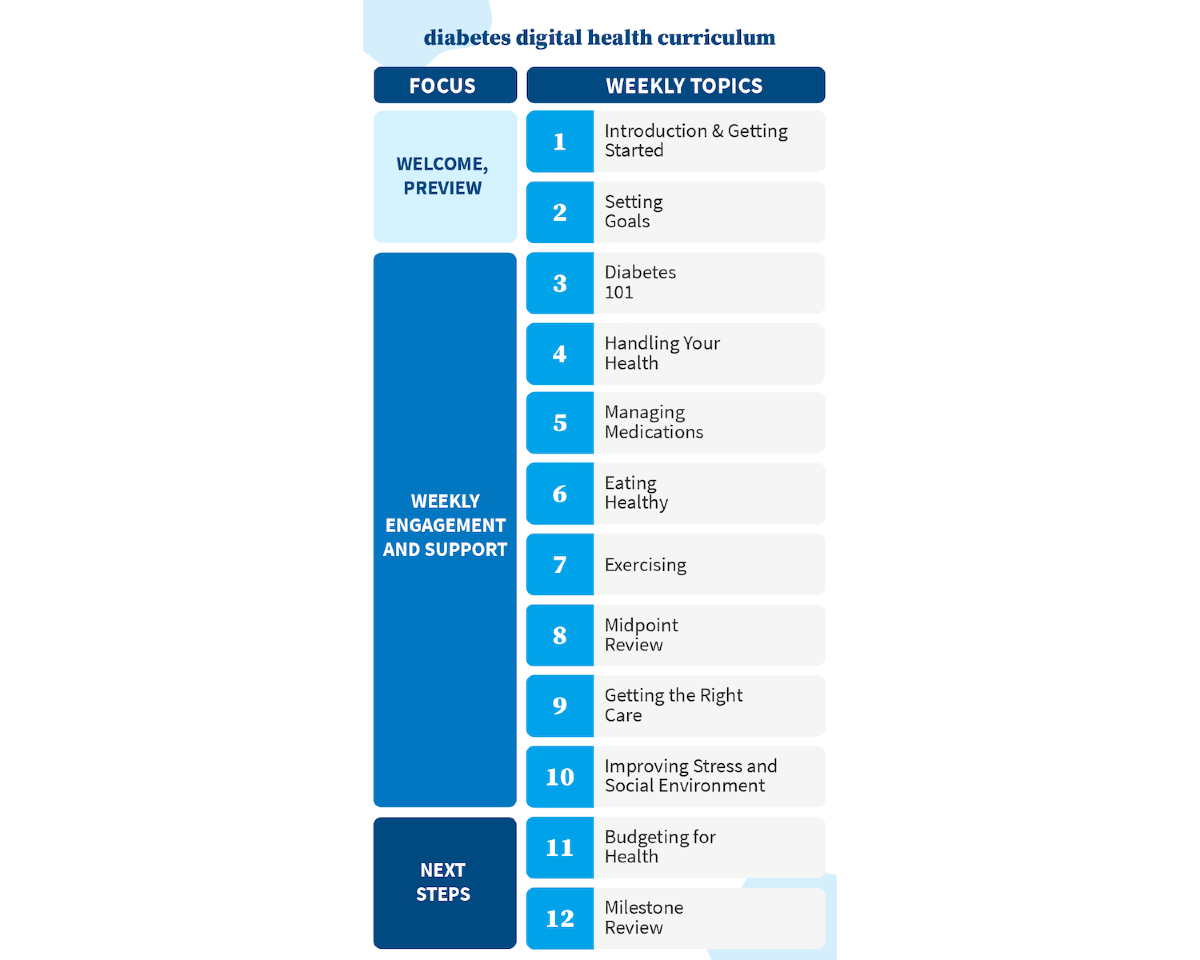
Diabetes curriculum.

The digital health coaching intervention represented in this study was carried out over 12 weeks. During that time, all participants were exposed to the same core intervention process and modular diabetes curriculum, as outlined in Figures 1 and 2. Minor customizations of supplemental content, such as links to medication discount services and referrals to community resources, were provided by coaches in accordance with a defined framework to address individual concerns or health goals of the participants.

At enrollment, potential participants were assigned a personal health coach, who remained their coach every week to promote a trusting interpersonal relationship. A preintervention introductory phone call was used to provide a detailed program overview and establish the participant’s communication preference (ie, phone calls and SMS text messaging or phone calls and email). Once enrolled, participants were asked to complete an online preintervention survey assessment to collect baseline patient-reported outcome metrics, set an overarching health goal for their 12-week experience, and complete a habit tracker to assess their weekly progress. Participants then received a standard “pack” of materials in the mail designed to facilitate ongoing self-management. The pack included an exercise stretch band, a goal magnet, and a journal for tracking symptoms and personal goals.

Between weeks 1 and 11, participants received a standard diabetes curriculum in addition to targeted supplementary coaching related to their goals. Before each weekly session, participants were asked to review video-based micro-lessons and supplemental educational resources. During the weekly call, health coaches answered questions, addressed areas of need, and helped participants identify an achievable daily goal to be tracked between calls (eg, exercise for 30 minutes). These daily goals, referred to as “tiny steps,” were used to reinforce desired behavioral and psychosocial changes and were reflected in 3 to 5 scheduled weekly SMS text messaging or email nudges. SMS text messaging and email nudges included reminders to prioritize goals and supplemental resources to help support goal attainment (eg, educational videos, recipes, worksheets, and articles). This process was repeated weekly as participants completed the curriculum. At week 12, coaches and participants reviewed goal progress and discussed strategies for sustainability. Throughout the 12-week process, coaches followed detailed call guidelines, which provide structure but not scripting, to deliver a modular diabetes curriculum that was provided to all participants. The standard curriculum covers symptom management, complication/comorbidity prevention, medication management, healthy eating, physical activity, patient-provider communication, gap elimination in care, psychosocial wellness (stress, sleep, and social support), and budgeting for health and sustainability (Figure 2).

### Participant Enrollment

Participation was voluntary, and individuals could withdraw from the program at any time with no risks or penalties. Participants were recruited between July 2016 and March 2020 from a variety of channels, including employer benefit programs, health plans, nonprofit partners, and provider referrals. Enrollment methods varied by channel. Customized emails and webpages were used to recruit participants through employer benefit programs, health plans, and nonprofit partners, while printable flyers were used by referring providers.

All potential participants were given the option to enroll in the intervention online or by phone. Individuals who opted for electronic enrollment completed an online interest form to determine eligibility, which has been defined below. Eligible individuals were emailed a program information sheet and a unique link to an electronic informed consent form, including a comprehensive description of rights, obligations, and risks. Telephone enrollment followed a similar process carried out by a program coordinator. The coordinator provided an oral description of the program, verified eligibility, and read the consent form over the phone. Eligible participants consented verbally.

The eligibility criteria for program participation included confirmed T2DM diagnosis, age 18 years or older, and ability to read, speak, and consent in English. Program participants were included in the study cohort if they were considered active at 12 weeks and completed all baseline and follow-up metrics included in the study (Table 1) [46,47]. Participants were defined as active if they completed at least one communication, including phone calls, SMS text messaging replies, and survey responses, in the previous 14 days.

Health coaching was provided at no cost to participants, with expenses covered by respective referral partners or through an external research grant. Data collected throughout the enrollment and coaching processes were anonymized, aggregated, and stored in a Health Insurance Portability and Accountability Act–compliant platform for future use in system and program optimization. Subsequently, no institutional review board approval was sought for this retrospective real-world analysis.

**Table 1:** Study measures and instruments.

Outcome and measurement			Instrument		Description		Scale or threshold	
			
**Clinical outcome**								
	HbA_1c_^a^	Self-reported HbA_1c_		Average blood sugar level (%) over the past 2-3 months		HbA_1c_ < 7%: low risk 7% ≤ HbA_1c_ ≤ 9%: moderate risk HbA_1c_ >9%: high risk		
	Weight	Self-reported weight in lbs		N/A^b^		N/A		
	BMI	Self-reported weight and height		BMI = 703 × weight (lbs)/height (in)^2^		BMI ≤18.5: underweight 18.5 < BMI ≤ 24.9: healthy 25 < BMI ≤ 29.9: overweight BMI ≥30: obese		
**Patient-reported outcome**								
	GPH^c^	PROMIS^d^ Global Health Short-Form v1.2 [46]		Physical health score is determined using Q3, Q6, Q7, and Q8		US average = 50 (SD 10) A higher value means better health		
	GMH^e^	PROMIS Global Health Short-Form v1.2 [46]		Mental health score is determined using Q2, Q4, Q5, and Q10		US average = 50 (SD 10) A higher value means better health		
	DDS-2^f^	Diabetes Distress Scale-2 [47]		Total DDS-2 score is determined by averaging scores across two items		DDS-2 score <2.0: little or no distress 2.0 ≤ DDS-2 score ≤ 3.0: moderate distress DDS-2 score >3.0: high distress		

^a^HbA_1c_: hemoglobin A_1c_.

^b^N/A: not applicable.

^c^GPH: Global Physical Health.

^d^PROMIS: Patient-Reported Outcomes Measurement Information System.

^e^GMH: Global Mental Health.

^f^DDS-2: Diabetes Distress Scale-2.

### Participant Characteristics and Process Measures

Demographic data, including participant gender, race, age, and state of residence, were collected during enrollment. Participant engagement was quantified by measuring the number and duration of completed weekly coaching calls and the number of opened digital nudges including micro-lessons and supplemental educational resources.

### Outcome Measures

To assess change from preintervention to postintervention, survey data were collected electronically via participant self-report at enrollment (baseline) and completion (12 weeks). Surveys were voluntary and no incentives were provided. All surveys, totaling six pages, were delivered on the same schedule for all participants via the preferred communication method established by the patient, typically through email or SMS text messaging. At the time of survey delivery, participants received a unique link tied to their individual identifier. The link expired once the survey was complete, therefore eliminating the chance of duplication.

A summary of study measures and instruments is found in Table 1. Self-reported HbA_1c_ was used to estimate changes in blood glucose levels. While not as reliable as lab-based measurements, self-reported HbA_1c_ values are commonly used in real-world practice and have been shown to be reliable within half of a percentage point and reflect lab-based values over 75% of the time [48]. BMI, an indicator of weight status, was calculated using self-reported weight and height measurements [49]. HbA_1c_, weight, and BMI calculations were tested across multiple test patients prior to implementation. Two validated web-based surveys were used to measure program impact on physical and mental health.

Overall physical and mental health were assessed using the 10-item Patient-Reported Outcomes Measurement Information System (PROMIS) Global Health Short-Form questionnaire version 1.2 [46], and diabetes-related distress was measured using the Diabetes Distress Scale-2 (DDS-2) [47]. The PROMIS Global Health short form measures health-related quality of life across the physical and mental domains, which are relevant to a variety of chronic conditions [46]. A Global Physical Health (GPH) score was determined using results from questions 3, 6, 7, and 8 pertaining to perceived physical health, physical ability, fatigue, and pain. A Global Mental Health (GMH) score was determined using results from questions 2, 4, 5, and 10 pertaining to overall mental health, including perceived quality of life, mood, satisfaction, and emotional problems. The GPH and GMH were normalized to a score of 50 (±10 for one standard deviation) to represent the average score for a person in the United States, with higher scores reflecting better physical and mental health. DDS-2 is a two-item instrument designed to assess emotional burden related to diabetes and diabetes regimen distress in a clinical setting. Sources are reflected in two subscales, and an overall DDS-2 score is determined by averaging scores across subscales, with higher scores (>3.0) reflecting high distress [47].

### Statistical Analysis

We conducted a retrospective cohort analysis to examine the impact of the digital health coaching program in a convenience sample of adults with T2DM stratified by baseline HbA_1c_. Glycemic risk categories were defined using baseline HbA_1c_ values and were designed to align with participant risk for vascular complications and all-cause mortality. In accordance with the ADA *Standards of Care* [37] and Cavero-Rendondo et al [50], an HbA_1c_ value less than 7% was considered low risk and an HbA_1c_ value greater than 9% was considered high risk. For the purpose of this study, an HbA_1c_ value between 7% and 9% was considered moderate risk.

All statistical analyses, descriptive and correlational, were conducted using STATA 16 statistical software (StataCorp). Descriptive statistics were performed for all primary variables to measure baseline sample characteristics, participant engagement, and change across outcome metrics. Means and standard deviations were calculated for continuous variables, while frequencies and percentages were determined for categorical variables.

The absolute difference in each outcome metric (HbA_1c_, BMI, GPH, GMH, and DDS-2) was calculated using preintervention to postintervention values for each cohort and for the overall sample population. One-sample paired *t* tests were performed to evaluate the significance of this difference, with the null hypothesis being no change in HbA_1c_, BMI, or PROMIS scores. All tests were conducted at the significance level of α=.05. No weighting or matching methods were performed to adjust for a nonrepresentative sample. Incomplete surveys were excluded from the analysis.

## Results

### Participant Characteristics and Process Measures

**Figure 3:**
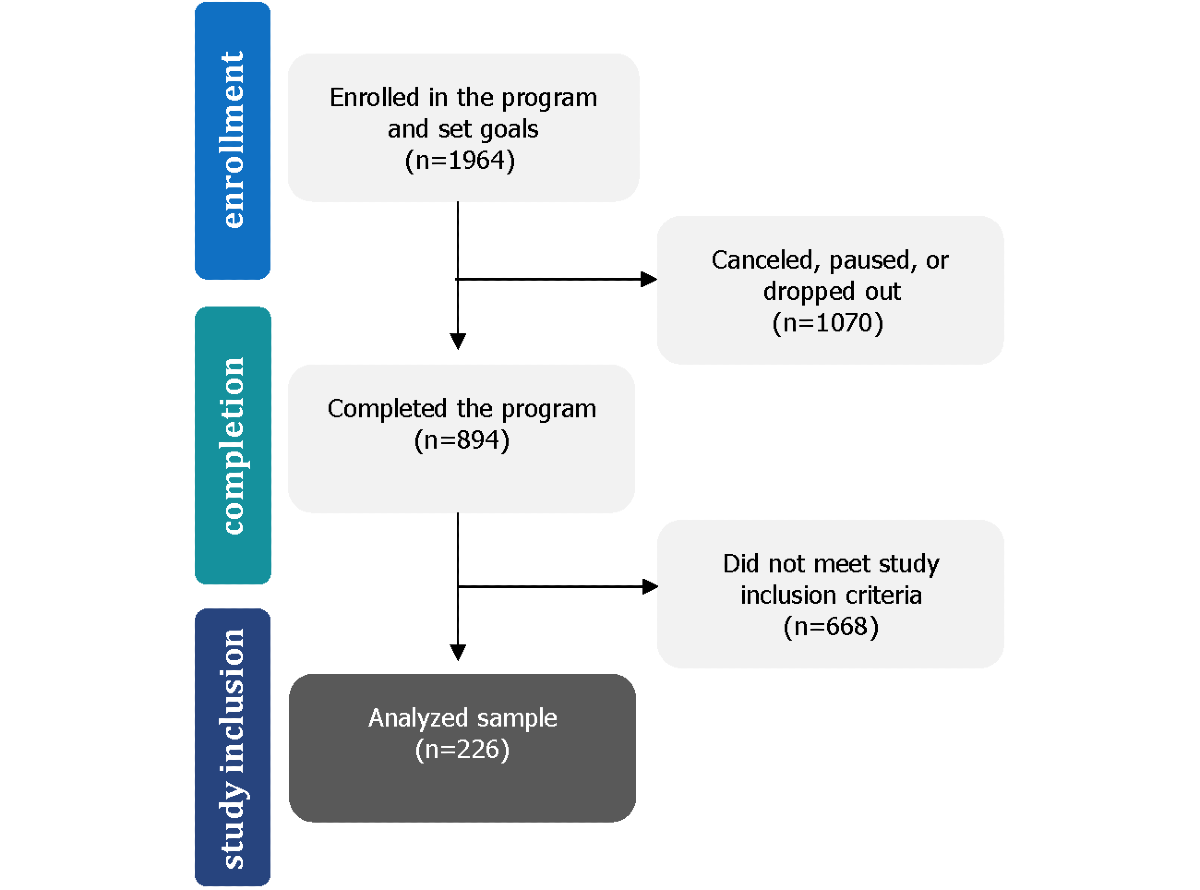
Study flow chart.

Of the 1964 participants who enrolled in the program, 1070 (54.5%) did not meet our definition for active participation at 12 weeks. This is consistent with attrition rates for other digital diabetes interventions reflected in published observational studies [51]. Of those participants remaining in the sample, 668 (34.0%) failed to complete the surveys defined in the inclusion criteria for this study, which were optional for completing the program. Attrition includes individuals who enrolled in the program but did not complete a first call or who discontinued participation prior to the completion of the 12-week program. Of the 894 participants who completed the program, 226 (25.3%) had complete HbA_1c_ data sets at baseline and follow-up, and were subsequently included in the study (Figure 3).

Descriptive statistics for the study sample (n=226) are reported in Table 2. The sample population was 71.7% (162/226) female, and the majority of participants were white (139/226, 61.5%) or black (77/226, 34.1%). The mean age of participants at enrollment was 59.1 years (SD 9.23). The most common referral sources were providers (73/226, 32.3%), health-sponsored plans (54/226, 23.9%), and employers (43/226, 19.0%), drawing participants from 26 states within the continental United States.

Most patients were considered overweight or obese, with a mean BMI of 35.8 (SD 7.79) at enrollment [49]. Baseline HbA_1c_ levels ranged from 6.5% to 17.6%, with a mean self-reported baseline HbA_1c_ of 8.17% (SD 1.55%). For the purpose of glycemic risk stratification and subgroup analysis, 18.6% (42/226) of participants were considered low risk (HbA_1c_ <7.0%), 61.1% (138/226) were considered moderate risk (7% ≤ HbA_1c_ ≤ 9%), and 20.3% (46/226) were considered high risk (HbA_1c_ >9.0%).

**Table 2:** Patient and clinical demographics at enrollment (N=226).

Demographic			Value, n (%)	
**Gender**				
	Female	162 (71.7%)		
	Male	64 (28.3%)		
**Race**				
	White	139 (61.5%)		
	Black	77 (34.1%)		
	Other/multiracial	10 (4.4%)		
**Enrollment type**				
	Provider	73 (32.3%)		
	Payer	54 (23.9%)		
	Employer	43 (19.0%)		
	Pharmaceutical	31 (13.7%)		
	Self-enrollment	25 (11.1%)		
**HbA_1c_^a^ status at baseline**				
	Low risk (HbA_1c_ <7%)	42 (18.6%)		
	Moderate risk (7% < HbA_1c_ < 9%)	138 (61.1%)		
	High risk (HbA_1c_ >9%)	46 (20.3%)		

^a^HbA_1c_: hemoglobin A_1c_.

On average, participants received 5.75 multichannel communications per week from their coaches over the course of the 12-week engagement. Weekly calls with personal health coaches lasted an average of 14.53 minutes per call, and participants received an average of 3.75 digital nudges and one digital educational resource via SMS text messaging or email per week.

### Outcome Measures

**Figure 4:**
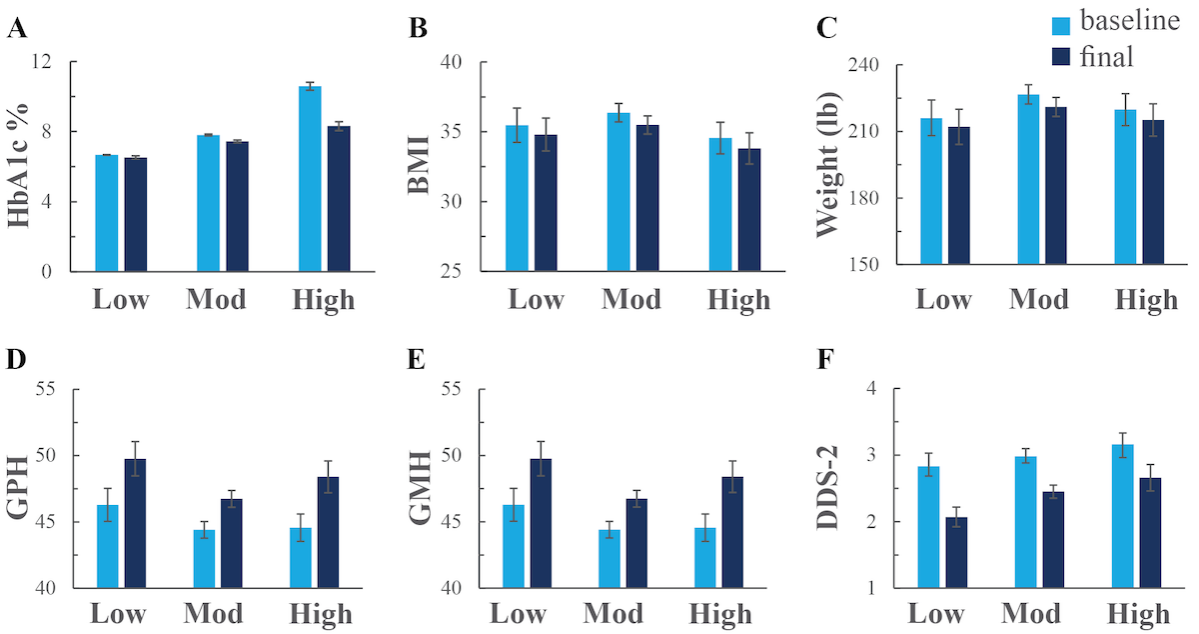
Clinical and patient-reported outcomes before and after digital health coaching across glycemic risk groups including (A) blood glucose levels according to hemoglobin A_1c_ (HbA_1c_), (B) weight status according to BMI, (C) weight in pounds, (D) physical health according to Patient-Reported Outcomes Measurement Information System (PROMIS) Global Physical Health (GPH), (E) mental health status according to PROMIS Global Mental Health (GMH), and (F) distress according to the Diabetes Distress Scale-2 (DDS). Self-reported measures were collected at enrollment (baseline) and upon completion of the 12-week program (final) for participants with low, moderate, and high baseline HbA_1c_ levels, reported as mean ± standard error of the mean for each cohort. Mod: moderate.

Across the total sample, the mean HbA_1c_ decreased from 8.17% (SD 1.55%) to 7.44% (SD 1.33%), resulting in a clinically relevant reduction of 0.73 percentage points (*P*<.001) (Table 3, Figure 4A). Reductions in blood glucose levels were observed in all glycemic risk groups, though a greater reduction was observed in groups with higher baseline HbA_1c_ levels (Table 3, Figure 4A). High-risk participants with an HbA_1c_ ≥9% reported the largest improvements. Their HbA_1c_ levels decreased by 2.28 percentage points or 21% compared with baseline (*P*<.001). Moderate-risk participants reported a 0.37 percentage point decrease or a 5% reduction from baseline (*P*<.001). Low-risk participants maintained glycemic control with no significant change in HbA_1c_ from baseline to follow-up.

**Table 3:** Primary study outcomes.

Measurement^a^			Baseline value, mean (SD)		Final value, mean (SD)		Difference, Δ (%)		*P* value^b^	
**HbA_1c_^c^ (%)**										
	Low risk	6.67 (0.13)		6.52 (0.56)		−0.15 (−2.25)		.09		
	Moderate risk	7.81 (0.59)		7.44 (0.93)		−0.37 (−4.74)		<.001		
	High risk	10.59 (1.57)		8.31 (1.74)		−2.28 (−21.43)		<.001		
	Overall	8.17 (1.55)		7.44 (1.23)		−0.73 (−8.94)		<.001		
**Weight (lbs)**										
	Low risk	216.05 (52.31)		212.13 (51.58)		−3.92 (−1.81)		.003		
	Moderate risk	226.70 (51.54)		221.09 (50.48)		−5.61 (−2.47)		<.001		
	High risk	219.83 (49.25)		215.19 (49.23)		−4.64 (−2.11)		.002		
	Overall	223.21 (51.19)		218.23 (50.35)		−5.09 (−2.28)		*<*.001		
**BMI**										
	Low risk	35.47 (7.95)		34.80 (7.62)		−0.67 (−1.89)		.002		
	Moderate risk	36.36 (7.77)		35.48 (7.69)		−0.88 (−2.42)		*<*.001		
	High risk	34.55 (7.68)		33.81 (7.58)		−0.74 (−2.14)		.001		
	Overall	35.83 (7.79)		35.01 (7.66)		−0.82 (−2.29)		<.001		
**Global Physical Health score**										
	Low risk	46.28 (8.06)		49.76 (8.36)		3.48 (7.52)		.007		
	Moderate risk	44.40 (7.28)		46.74 (7.44)		2.34 (5.27)		*<*.001		
	High risk	44.56 (6.98)		48.40 (8.10)		3.84 (8.62)		.001		
	Overall	44.78 (7.34)		47.64 (7.81)		2.86 (6.39)		<.001		
**Global Mental Health score**										
	Low risk	48.85 (7.15)		52.02 (8.86)		3.17 (6.49)		.01		
	Moderate risk	48.19 (7.27)		50.02 (7.91)		1.83 (3.80)		.006		
	High risk	46.11 (7.84)		49.41 (7.86)		3.3 (7.16)		.001		
	Overall	47.88 (7.39)		50.27 (8.09)		2.39 (4.99)		<.001		
**Diabetes Distress Scale-2 score**										
	Low risk	2.83 (1.29)		2.07 (0.95)		−0.76 (−26.86)		.005		
	Moderate risk	2.98 (1.37)		2.45 (1.15)		−0.53 (−17.78)		<.001		
	High risk	3.16 (1.17)		2.66 (1.35)		−0.5 (−15.82)		.05		
	Overall	2.99 (1.31)		2.42 (1.17)		−0.57 (−19.06)		<.001		

^a^Categorized by baseline HbA_1c_ as follows: low risk, HbA_1c_ <7%; moderate risk, 7% ≤ HbA_1c_ ≤9%; high risk, HbA_1c_ >9%.

^b^*P* value calculated using the chi-square test with comparison of the relative value to baseline.

^c^HbA_1c_: hemoglobin A_1c_.

Overall, participants who completed the program lost weight, thereby reducing their BMI. The weight decreased by an average of 5.09 pounds (*P*<.001), and BMI decreased by an average of 0.82 points (*P*<.001). The trend observed for reductions in weight and BMI within glycemic risk groups was different from the trend observed for reductions in HbA_1c_ levels (Table 3, Figure 4B-C). In this case, the moderate-risk cohort saw the greatest change in BMI with a 0.88 point reduction (*P*<.001), followed by the high-risk cohort with a 0.74 point reduction (*P*<.001) and the low-risk cohort with a 0.67 point reduction (*P*=.002).

Improvements in physical and mental health, as measured by PROMIS scores, were also significant across all groups (Table 3, Figure 4D-E). Across cohorts, the GPH score significantly increased by 6.4% to a final score of 47.64 (SD 7.81; *P*<.001), and the GMH score increased by 5% to a final score of 50.27 (SD 8.09; *P*<.001). Participants with high glycemic risk showed the greatest improvement in physical and mental health scores, with increases of 3.84 points (*P*<.001) for GPH and 3.3 points (*P*=.001) for GMH.

Improvements in diabetes distress were also significant across all groups (Table 3), where the average DDS-2 score improved by 0.57 (from 2.99 [SD 1.31] to 2.42 [SD 1.17]). For this metric, trends by glycemic risk did not align with the other patient-reported outcomes; the greatest improvement was observed for the low-risk cohort. An improvement of 0.76 (*P*=.005) (from 2.99 to 2.42) was observed for the low-risk cohort compared with an improvement of 0.5 (*P*=.053) (from 3.16 to 2.66) for the high-risk cohort.

## Discussion

### Principal Findings

This retrospective study of real-world data indicated that participants with T2DM who completed the multichannel digital health coaching program and provided complete HbA_1c_ data sets achieved improved glycemic control and body weight, in addition to positive changes in their physical and mental health status. Cohort analysis revealed that individuals with the highest glycemic risk (HbA_1c_ >9% at enrollment) achieved the greatest level of change in all clinical and patient-reported outcomes, except for weight loss (BMI). The findings reported here are consistent with the study hypothesis and align with emerging evidence on digital health coaching intervention outcomes [20-32].

Prior randomized controlled trials and observational studies have established the efficacy of digital health coaching to improve glycemic control and encourage weight loss among individuals with T2DM [25-27]. The average weight loss and HbA_1c_ reduction in the overall cohort was consistent with values reported in response to similar digital interventions. However, comparatively few studies reported specific outcomes for individuals with a high glycemic risk [25,26]. To our knowledge, this is the first study to evaluate the differential impact of digital health coaching for cohorts stratified based on baseline glycemic risk.

This study showed that patients with elevated blood glucose (HbA_1c_ >9%) reduced their HbA_1c_ levels by 2.28 points (*P*<.001). This degree of HbA_1c_ reduction is greater than many values reported in existing literature on the impact of similar digital health coaching interventions [25,26,52]. While some studies have shown comparable improvements for high-risk participants, these interventions have typically relied upon clinicians for management and/or education [30]. This result observed in real-world self-reported HbA_1c_ levels is promising, though it should be confirmed with laboratory measurements of HbA_1c_ in a larger randomized efficacy trial. If verified, these results could imply that digital health coaching for this high-risk population can have a significant impact on diabetes outcomes beyond glycemic control alone. For every 0.9% decrease in HbA_1c_, patients benefit from a 10% decrease in diabetes-related mortality, a 25% reduction in microvascular complications, and a 6% reduction in overall mortality [53].

In this study, we also noted an average weight loss of 5.09 pounds and an average reduction in BMI of 0.82 points (2.29%; *P*<.001) across all cohorts. Weight loss is associated with substantial health benefits for obese patients, where losing 5% to 10% of body weight reduces the risk of cardiovascular comorbidities [54]. However, even modest weight loss in the range of 2% to 5% can provide clinically meaningful reductions in fasting blood glucose for obese patients with diabetes [54]. In contrast to the trend observed in HbA_1c_ level reduction, the moderate-risk cohort lost more weight and reduced BMI more than the high-risk cohort. Importantly, this may be explained by differences in average cohort weight at enrollment. The moderate-risk cohort reported the highest baseline BMI (36.36) at enrollment, which may indicate that baseline BMI is a stronger predictor of weight loss than baseline HbA_1c_. These findings are important to consider in future digital health coaching research given the role of weight loss in improved cardiometabolic outcomes for individual with T2DM [54,55].

Moreover, findings from this study suggest that individuals at high baseline glycemic risk may experience the greatest benefit in overall physical and mental health in response to digital health coaching. Trends in PROMIS GPH for high-risk participants showed the most notable impact when compared to individuals with lower baseline HbA_1c_. At the 12-week follow-up, the high-risk group’s GPH score improved by 3.84 points (*P*=.001) compared with 2.86 points (*P*<.001) for the overall population. Similar trends were observed for PROMIS GMH scores.

At baseline, the average patient-reported GMH score was 47.89, which is below the national average of 50.00 [46]. For high-risk patients, that average was 46.11, indicating more room for improvement. After the intervention, the average score across cohorts was 50.27 or slightly above the national average. High-risk participants reported an improvement of 3.3 points (*P*<.001). If sustained, the positive shift for this cohort could drive improvements to physical health given the relationship between mental health and glycemic control [5,6].

Change in distress from preintervention to postintervention was significant across all cohorts (*P*<.001). The high-risk group demonstrated the greatest need for change, with a baseline score of 3.16, indicating high distress, compared with a baseline score of 2.83 for the low-risk group, indicating moderate distress. High distress scores are associated with negative diabetes outcomes, including high HbA_1c_, low self-efficacy, and poor diet [56]. The reductions in diabetes-related distress reported here are larger than those reported after a 12-month mobile diabetes intervention, which may be explained by differences in distress levels at baseline [29]. Interestingly, the trend observed in overall mental health status did not translate to diabetes distress levels, and instead, those individuals with the lowest baseline glycemic risk showed the greatest improvement, reducing their distress levels to low or nearly no distress. This indicates that even those groups with well-controlled HbA_1c_ at baseline can benefit from the program.

Overall, these findings indicate that higher glycemic risk patients may have a greater need for mental and physical health monitoring and, with the exception of reducing distress, may also have the greatest potential for experiencing a positive impact from multichannel digital diabetes health coaching.

### Limitations of the Study

Although this study provides valuable insights into the real-world application of digital diabetes health coaching and specifically who may benefit most, the reported findings are limited by the real-world cohort study design. Most notably, no control group was available to compare interventions to standard of care practices. As a result, we cannot rule out that the observed improvements were due to factors unrelated to the digital health coaching program. Furthermore, patients were not incentivized using the same tools typical of a research trial (eg, financial incentives, trust in clinicians, and access to better health care), which can impact attrition rates and data collection [57]. While these issues are well documented in diabetes supportive care programs [58], they resulted in incomplete data sets and a subsequently limited cohort size. Finally, self-reported HbA_1c_ and BMI were not confirmed by laboratory data. As a result, while our findings are consistent with results presented in other studies, there were too many uncertainties to make definitive conclusions. However, we believe these results and observations can be leveraged to inform the design of future prospective efficacy studies investigating baseline HbA_1c_ and BMI as possible mediators of the impact of digital health coaching.

### Conclusions

This real-world analysis provides valuable insights on the impact of digital health coaching on T2DM control for participant glycemic risk. While program completion was associated with improved patient-reported outcomes for the average participant, participants with a high HbA_1c_ level at baseline saw the greatest improvement in glycemic control and overall physical and mental health. Further research is warranted to fully understand the differential impact of multichannel digital health coaching support for patients with increasing HbA_1c_.

### Perspectives and Implications

To our knowledge, this is the first real-world study to examine the clinical predictors of program outcomes for diabetes patients who participate in digital health coaching. These findings are relevant to many stakeholders, including clinicians, health systems, employers, and payers, who must decide which patients to refer to such a program. As this type of low-cost and accessible intervention continues to scale nationally, informed referral strategies will become increasingly important.

## Abbreviations

ADA: American Diabetes Association
DDS-2: Diabetes Distress Scale-2
GMH: Global Mental Health
GPH: Global Physical Health
HbA_1c_: hemoglobin A_1c_
PROMIS: Patient-Reported Outcomes Measurement Information System
T2DM: type 2 diabetes mellitus
